# Recognition of heparan sulfate by clinical strains of dengue virus serotype 1 using recombinant subviral particles

**DOI:** 10.1016/j.virusres.2013.04.017

**Published:** 2013-05-22

**Authors:** Charlermchai Artpradit, Luke N. Robinson, Boris K. Gavrilov, Troy T. Rurak, Mathuros Ruchirawat, Ram Sasisekharan

**Affiliations:** a Program in Applied Biological Sciences: Environmental Health, Chulabhorn Graduate Institute, Bangkok, Thailand; b Department of Biological Engineering and Koch Institute for Integrative Cancer Research, Massachusetts Institute of Technology, Cambridge, MA, United States; c Laboratory of Environmental Toxicology, Chulabhorn Research Institute, Bangkok, Thailand

**Keywords:** Dengue, Heparan sulfate, Recombinant subviral particle, Receptor

## Abstract

Dengue is the most important arthropod-borne viral disease in humans, with an estimated 3.6 billion people at risk for infection and more than 200 million infections per year. Identification of the cellular receptors for dengue virus (DV), the causative agent of dengue, is important toward understanding the pathogenesis of the disease. Heparan sulfate (HS) has been characterized as a DV receptor in multiple model systems, however the physiological relevance of these findings has been questioned by observations that flaviviruses, including DV, can undergo cell culture adaptation changes resulting in increased binding to HS. It thus remains unclear whether HS is utilized by clinical, non-cell culture-adapted strains of DV. To address this question, herein we describe a set of methodologies using recombinant subviral particles (RSPs) to determine the utilization of HS by clinical strains of DV serotype 1 (DV1). RSPs of clinically isolated strains with low cell culture passage histories were used to study HS interaction. Biochemically characterized RSPs showed dose-dependent binding to immobilized heparin, which could be competed by heparin and HS but not structurally related glycosaminoglycans chondroitin sulfate A and hyaluronic acid. The relevance of heparin and HS biochemical interactions was demonstrated by competition of RSP and DV binding to cells with soluble heparin and HS. Our results demonstrate that clinical strains of DV1 can specifically interact with heparin and HS. Together, these data support the possibility that HS on cell surfaces is utilized in the DV-human infection process.

## 1. Introduction

Dengue is a mosquito-borne disease of increasing threat to public health in many tropical and subtropical regions around the world and shows a growing geographical footprint. Dengue causes significant morbidity and mortality worldwide, with approximately 230 million infections and 21,000 deaths annually, along with substantial economic costs and burden to healthcare systems (Gubler, 2012). Infection with DV results in a wide spectrum of clinical manifestations, ranging from asymptomatic presentation, to flu-like febrile disease termed dengue fever (DF), and potentially to more severe and life-threatening forms termed dengue hemorrhagic fever (DHF) and dengue shock syndrome (DSS) (Halstead, 2007; Harris et al., 2000). With no approved specific treatment or vaccine, dengue is a significant and growing global public health issue (Farrar et al., 2007; Miller, 2010; Simmons et al., 2012).

DV is composed of four closely related serotypes, termed 1–4, and is a member of the *Flavivirus genus*, along with other important human pathogens including tick-borne encephalitis virus (TBEV), West Nile virus (WNV), and Japanese encephalitis virus (JEV). Structurally, DV is an enveloped positive single-stranded RNA virus. The 10.7 kb RNA genome of DV is comprised of three structural genes, which encode for capsid (C), premembrane (prM), and envelope glycoprotein (gpE), as well as seven non-structural genes. The mature virus particle structure is approximately 50 nm in diameter, with the outer surface composed of 90 gpE homodimers arranged with icosahedral symmetry (Kuhn et al., 2002). During the natural flavivirus infection process, subviral particles (SPs) in addition to virions, are generated (Corver et al., 2000; Russell et al., 1980). SPs are characterized as non-infectious particles generally about 30 nm in diameter containing prM/M and gpE surface proteins on a lipid bilayer but are devoid of capsid and an RNA genome (Allison et al., 2003). SPs can be produced recombinantly by the co-expression of prM and gpE genes in mammalian cells (Holmes et al., 2005; Hsieh et al., 2008; Liu et al., 2010; Schalich et al., 1996). RSPs have similar envelope protein presentation, hemagglutination and fusion activity to infectious virus particles (Corver et al., 2000; Schalich et al., 1996). As a result, RSPs have been exploited as an alternative to infectious DV for diverse applications, including vaccine development, delivery systems, and antigens for diagnostics as reviewed elsewhere (Shang et al., 2012).

Like many other enveloped viruses, DV infects host cells first by recognizing and binding receptor(s) on cell surfaces, however no clear consensus of the receptor identities for DV has emerged. Multiple putative receptors found on different cell types have been proposed, including DC-SIGN on primary human monocyte-derived dendritic cells and Langerhan cells; mannose receptor on macrophages; Fcγ receptor on various immune cells; phosphatidylserine-receptor TIM and TAM proteins (Cabrera Hernandez and Smith, 2005; Hidari and Suzuki, 2011); and HS on multiple cell lines, including human hepatoma cell (Huh7), Vero cells, CHO cells and BHK-21 cells (Chen et al., 1997; Germi et al., 2002; Hilgard and Stockert, 2000; Kato et al., 2010; Lin et al., 2002), as well as other cell types (Dalrymple and Mackow, 2011; Okamoto et al., 2011; Thaisomboonsuk et al., 2005; Thepparit et al., 2004). HS is a member of the glycosaminoglycan (GAG) family of glycans and is extensively found on the surface of mammalian cells. Structurally, it is composed of a repeating disaccharide unit of glucuronic acid linked to *N*-acetyl-glucosamine (Sasisekharan et al., 2006). HS chains have been shown to serve as attachment factors for a variety of microorganisms and viruses, including flaviviruses (Zhu et al., 2011). In multiple studies, HS has been shown to play a role in the DV infection process (Hilgard and Stockert, 2000; Martinez-Barragan and del Angel, 2001; Thepparit et al., 2004), suggesting that HS may indeed serve as a receptor or co-receptor for DV infection of host cells.

Other studies investigating the role of HS in DV infection have demonstrated that DV, when propagated under certain conditions *in vitro*, can undergo adaptive changes resulting in increased affinity for and utilization of HS for cell infection (Anez et al., 2009; Lee et al., 2006b; Prestwood et al., 2008). Indeed, similar studies with other flaviviruses, including TBEV and JEV, have also shown the potential of these viruses to undergo genetic changes upon cell culture propagation which also confer an increased HS binding phenotype (Lee and Lobigs, 2002; Mandl et al., 2001). These results call into question whether naturally circulating DV strains utilize HS as an attachment factor.

To address this question, we generated RSPs which encode prM–gpE sequences of four DV1 strains, two of which represent low-passage clinical isolates and two of strains extensively passaged in cell culture. Purified RSPs were biochemically and biophysically characterized for their integrity and similarity to dengue virions. Characterized RSPs of clinical DV isolates, in addition to adapted strains, were shown to bind immobilized heparin, and this interaction could be specifically competed with heparin and HS but not chondroitin sulfate and hyaluronic acid. HS interactions were further interrogated using a cell-based assay, in which RSPs of clinical and adapted strains were shown to bind Vero cells in a manner which could be competed for by heparin and HS. Similarly, Vero cell binding of a DV1 strain with an extensive passage history could also be competed for by heparin and HS, supporting that the findings with RSPs reflect infectious virus mechanisms. Collectively, these results support the notion that DV utilizes HS on cell surfaces as an attachment factor during the infection process.

## 2. Methods

### 2.1. Cell lines and viruses

Suspension human embryonic kidney 293 Freestyle (Freestyle 293) cells were purchased from Life Technologies and maintained in Freestyle 293 medium (Life Technologies). Vero and C6/36 cells were obtained from ATCC and cultured with DMEM/F12 supplemented with 10% FBS and EMEM supplemented with glutamine and 10% FBS, respectively (Life Technologies). *Spodoptera frugiperda* (Sf9) cells were purchased from ATCC, adapted to serum-free suspension culture, and maintained with BD BaculoGold medium (BD Bioscience). DV serotype 1 strain TH-Sman was purchased from ATCC and was grown in C6/36 cells.

### 2.2. gpE expression

Full length prM and 80% gpE (*i.e*., C-terminus 20% truncated) from DV1 strain FGA/NA d1d (Despres et al., 1998) was codon optimized and synthesized with a C-terminus 6X histidine tag (DNA 2.0). PrM-gpE was cloned using *XmaI* and *Bgl*II into PAcGP67a plasmid (BD Bioscience). Baculovirus of prM–gpE was generated following manufacturer’s instructions (BD Bioscience). Suspension Sf9 cells were infected with prM–gpE baculovirus, and conditioned medium was harvested 48 h post-infection and purified by FPLC using a HisTrap HP column (GE healthcare). The identity of gpE was confirmed by western blot and ELISA analyses probing with conformational-sensitive 4G2 and 1A1D2 antibodies, as described below.

### 2.3. RSP production

Codon optimized prM–gpE DNA sequences from all selected strains (Supplemental Table 1) were synthesized (DNA 2.0) and cloned into pcDNA 3.3 vectors (Life Technologies). Freestyle 293 cells were transiently transfected with purified plasmids using 25 kD linear polyethylenimine (Polyscience, Inc.,). After 5 days, culture supernatants containing RSPs were clarified at 3000 × *g* for 10 min and subsequently filtered through a 0.2 μm filter. RSPs were then pelleted by ultracentrifugation at 28,000 rpm for 4 h using rotor 32 Ti (Beckman Coulter). Pelleted RSPs were resuspended in PBS and subsequently purified by sucrose gradient (20–60%) ultracentrifugation. Sucrose solution was made in 20 mM HEPES pH 7.4 with 6 mL of each layer and an increment of 10% of sucrose. Ultracentrifugation was carried out at 28,000 rpm for 2.45 h. 1.5-mL fractions were harvested and assessed for the presence of gpE by western blot with 4G2, as described below. Fractions containing RSPs were further processed by dialysis and then concentrated using 100KD MWCO Amicon Ultra-15 centrifugal filter unit (Millipore). All steps were performed at 4 °C.

### 2.4. Anti-dengue monoclonal antibody (mAb) production

Flavivirus cross-reactive mouse mAb 4G2 was produced by HB-112 hybridoma cells, which were purchased and maintained as described by ATCC. mAb 1A1D-2 (Lok et al., 2008) was produced by Freestyle 293 transient transfection. Briefly, DNA sequences of 1A1D-2 heavy chain and light chain variable regions (PDB accession 2R69) were synthesized (DNA 2.0) and cloned into pcDNA 3.3 plasmids harboring human heavy chain and light chain constant region sequences, respectively. Both plasmids were transfected into Freestyle 293 cells, as described above. Both antibodies were purified by protein A chromatography (GE Healthcare).

### 2.5. Western blotting analysis

RSP samples were separated by 4–12% NuPAGE bis–Tris SDS-PAGE gel and transferred to nitrocellulose membrane (Life Technologies). Membrane was subsequently probed with 4G2 anti-flavivirus mAb (Kuwahara and Konishi, 2010) followed by goat anti-mouse IgG with conjugated HRP (Santa Cruz Biotechnology). The membrane was developed with ECL Advanced Western Blot Detection Kit (GE Healthcare) and visualized by gel documentation. The data were analyzed by Alpha View program (ProteinSimple).

For quantitative western blot analyses, five serial dilutions of purified gpE protein were included in each blot for generation of a standard curve. RSP samples were diluted appropriately to fit in the linear range of the standard curve. All samples were loaded into gels with equal volumes, and run as described above. To generate the standard curve, the intensity of gpE monomer was plotted against its corresponding concentration. The standard curve was plotted, and concentration was calculated using Alpha view software (ProteinSimple) (Supplemental Fig. 1).

### 2.6. Transmission electron microscopy (TEM)

RSP samples were first purified by immunoaffinity chromatography (IAC) using 4G2 mAb coupled to CNBr-resin. The purified RSPs were buffer exchanged to PBS and stored at 4 °C for subsequent studies. RSPs were adsorbed onto FORMVAR carbon film coated copper grid (Electron Microscopy Sciences), washed drop-wise with 1 mL of PBS, then stained with 2% uranyl acetate in water. The stained grid was observed by transmission electron microscope at magnification of 150,000×.

### 2.7. MALDI mass spectrometry

Mass spectra were acquired on an Applied Biosystems 4800 Plus MALDI TOF/TOF Analyzer, equipped with a 355 nm Nd:YAG laser. 5 mg/mL solution of sinapinic acid in 1:1, acetonitrile/water with 0.1% TFA was used as matrix. On a standard 384-well stainless steel MALDI sample plate, 0.5 μL of IAC-purified RSPs samples and 0.5 μL of matrix solution was spotted and mixed in one well. Mass spectra were acquired under positive ion linear mode.

### 2.8. Heparin-binding ELISA

Heparin was conjugated to BSA using sodium acetate buffer pH 5.2 with the reductive amination agent sodium cyanoborohydride as described elsewhere (Hoffman et al., 1983). Heparin–BSA conjugate was purified from unconjugated BSA and heparin by TSKgel G3000SWxl (Tosoh Bioscience) size exclusion HPLC using PBS pH 6.5 as running buffer. Heparin–BSA (or BSA as a negative control) was coated overnight at 4 °C onto Maxisorb (Nunc) 96-well plates. The wells were blocked with 1% skim milk and then RSP serial dilutions in PBS were added. After 2 h, wells were washed and bound RSPs were detected with 1A1D-2 antibody followed by horseradish peroxidase conjugated rabbit anti-human IgG (Jackson immuno-research laboratory). TMB microwell peroxidase substrate (KPL Inc.) was used as a substrate for spectrophotometric detection. 100 μL of 1 N sulfuric acid was added to stop the reaction and absorbance at 450 nm was measured. For soluble GAG competition studies, the same procedure was used with one modification. Before applying RSPs to the plate containing adsorbed heparin–BSA, RSPs at 200 μg of gpE equivalence/mL were first preincubated for 2 h with equal volume of serially diluted GAGs in PBS. The mixtures were then applied to the heparin–BSA adsorbed plates and processed as described above.

### 2.9. RSP-Vero cell inhibition assay

Vero cells were seeded into 96-well plates and incubated overnight to allow attachment. RSPs at 6 μM equivalence of gpE in serum-free DMEM/F12 medium were mixed 1:1 (v:v) with heparin or HS in serum-free medium and incubated for 2 h. The RSP–glycan mixtures were then incubated for 4 h at 37 °C with seeded Vero cells. Unbound RSPs were removed by gently washing twice with 200 μL of serum-free medium. The cells were then fixed by addition of 3.7% formaldehyde for 20 min at room temperature. After washing twice with PBS, the cells were permeabilized using 0.1% Triton X-100 in PBS for 30 min. The permeabilized cells were washed 3 times with PBS then bound RSPs were detected with 100 μL of 1 μg/mL of 1A1D2 followed by 100 μL of 1:10,000 of rabbit anti-human IgG HRP conjugated (Jackson Immuno-Research Laboratory) in PBST. Bound RSPs were colorimetrically visualized as described previously in ELISA assay.

### 2.10. DV1 Vero cell inhibition assay

DV1 strain TH-Sman (50 focus-forming units) was incubated with heparin or HS in 100 μL of serum-free DMEM for 2 h and subsequently incubated with Vero cell monolayers in 24-well plates for 4 h at 37 °C to allow virus adsorption. The unbound virus was gently washed with 1 mL of serum-free DMEM, and an overlay of 2% serum-supplemented DMEM with 1% Aquacide (Merck Biosciences) was applied to wells. After 5 days, the overlay was removed and cells were fixed with buffered formalin. Cells were permeabilized by 0.1% Triton X-100 in PBS and virus foci detected by use of 1A1D-2 antibody.

## 3. Results

### 3.1. Strain selection and RSP expression

Multiple lines of evidence have indicated that DV utilizes HS as an attachment factor during *in vitro* infection of cells (Chen et al., 1997; Germi et al., 2002; Hilgard and Stockert, 2000; Kato et al., 2010; Lin et al., 2002; Martinez-Barragan and del Angel, 2001; Thepparit et al., 2004; Watterson et al., 2012). However, passaging of DV *in vitro*, under certain conditions, has been shown to augment DV infection sensitivity to HS and heparin (Anez et al., 2009; Lee et al., 2006b; Prestwood et al., 2008). It remains unclear whether natural circulating strains of DV, which have not undergone extensive *in vitro* passaging, also utilize HS as a receptor. To examine whether clinical DV isolates utilize HS, we first constructed RSPs representing four strains of DV1. Two strains, ThD1-0102 and BR-DF02 (designated L-ThD1 and L-BR, respectively), are of clinical, low-passage isolates, and two strains, 45AZ5 PDK-0/PDK-27 and TH-Sman (designated H-45AZ5 and H-TH, respectively) are characterized with more extensive cell culture passage histories (Supplemental Table 1). L-ThD1 was derived directly from an infected human sample without any passaging (Tang et al., 2010) and L-BR has been passaged 3 times in C6/36 cell line (Carvalho et al., 2010). Strain H-45AZ5, which has been shown to display a small plaque phenotype, a sign of increased heparin interaction, has undergone significant passaging as part of development as a vaccine candidate (Puri et al., 1997). Strain H-TH was passaged multiple times in suckling mice followed by passaging in C6/36 cells (Shiu et al., 1992).

DNA sequences encoding prM and gpE gene sequences from the four selected strains were synthesized and cloned into mammalian expression plasmids. RSPs were expressed by transient transfection of suspension HEK-293 cells with prM–gpE expression vectors and supernatants were purified by sucrose gradient ultracentrifugation, as described in Section 2.

### 3.2. RSP characterization

**Fig. 1:**
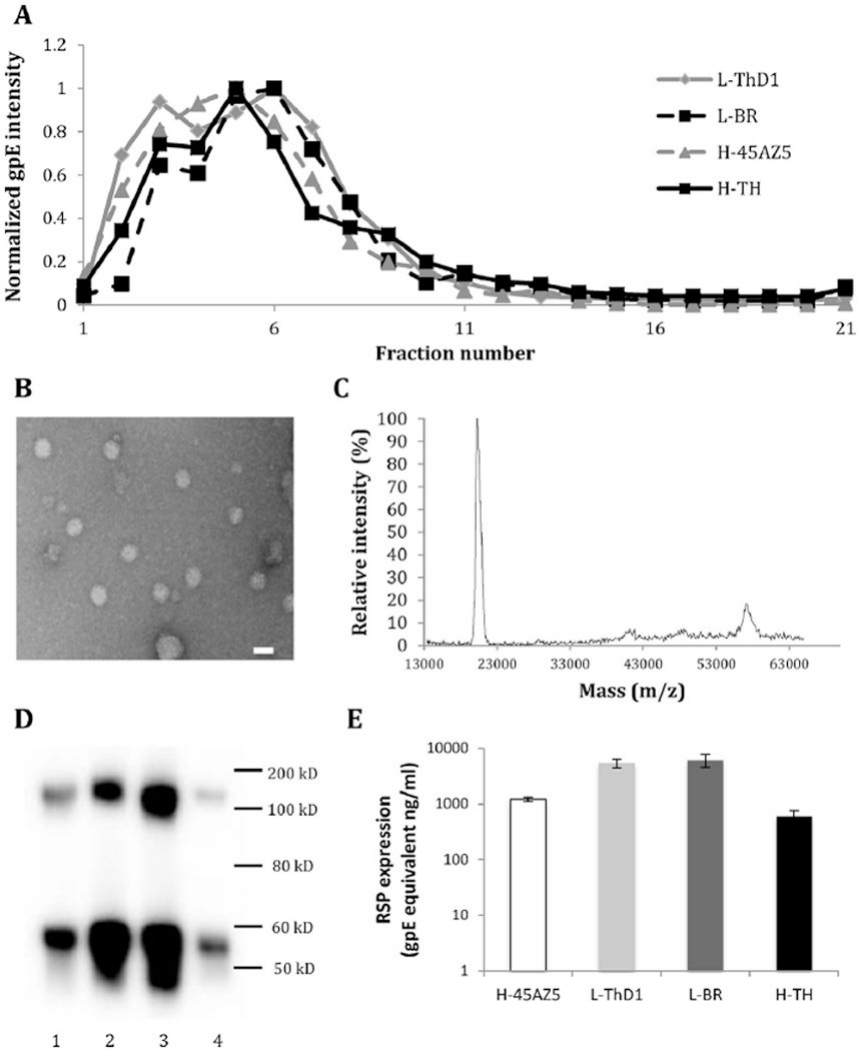
Biochemical and biophysical characterization of RSPs. (A) *Sucrose gradient analysis of RSPs*. Resuspended pellet from ultracentrifugation of transfected HEK 293 cell supernatant was overlaid onto 20–60% sucrose gradient. After ultracentrifugation for 2.5 h, 1.5 mL fractions were harvested from top to bottom and analyzed by western blot. The amount of gpE in each fraction was normalized by the fraction with maximum intensity of its strain. (B) *RSP structure by TEM*. IAC-purified RSP was adsorbed onto a carbon coated grid then stained with 2% uranyl acetate under magnification of 180,000×. Embedded bar represents 40 nm in length. (C) *MALDI-MS of RSPs*. IAC-purified RSP was analyzed using MALDI TOF/TOF analyzer equipped with 355 nm Nd:YAG laser using 5 mg/mL solution of sinapinic acid in 1:1, acetonitrile/water with 0.1% TFA as matrix. A representative mass spectrum is displayed. (D) *Western blot of RSP expression*. The expression of RSP of H-45AZ5 (lane 1), L-ThD1 (lane 2), L-BR (lane 3), and H-TH (lane 4) is displayed. Resuspended pelleted material from ultracentrifugation of transfected HEK 293 cell supernatant was analyzed by western blot probed with 4G2 anti-gpE antibody. (E) *Quantification of RSP expression levels*. The amount of gpE monomer from supernatant of transfected HEK 293 cells was quantified by western blot using 4G2 mAb. Purified recombinant gpE was included in the western blot as a standard protein to generate a standard curve. Error bars represent standard deviation.

Western blot of 1.5 mL fractions collected from sucrose gradient ultracentrifugation of RSPs (see Section 2) revealed the majority of gpE sedimented in fractions that correlated to 20–30% sucrose solution (Fig. 1A). Interestingly, two peaks of gpE after sucrose gradient ultracentrifugation were found for all strains, indicating the presence of two distinct populations, a finding consistent with Allison et al., which demonstrated two different sizes of flavivirus RSPs (Allison et al., 2003). To further investigate the particulate nature of the RSPs, samples were treated with Triton-X 100, a surfactant which disrupts lipid bilayer-containing particles, prior to sucrose gradient ultracentrifugation. Analysis of the fractions revealed gpE to be present in the top fraction (data not shown), indicating production of RSPs in intact particulate form (Schalich et al., 1996; Wang et al., 2009). To further assess the structural features of expressed RSPs, TEM of RSP samples was performed, which revealed the majority of RSPs to be round with the diameter of approximately 30 nm and a minority population with 50 nm diameter, findings consistent with previous studies (Allison et al., 2003; Liu et al., 2010; Schalich et al., 1996) (Fig. 1B and Supplemental Fig. 2). Results from ultracentrifugation and TEM analyses collectively demonstrate expression of intact RSPs with correct size.

To characterize protein constituents of RSPs, MALDI-MS of RSP samples was performed. Resultant spectra revealed three major peaks corresponding to molecular weights of approximately 8 kD (data not shown), 20 kD and 57 kD (Fig. 1C), which match closely to the theoretical molecular weights of M (8 kD), prM (20 kD) and glycosylated gpE (57 kD) (Allison et al., 2003; Schalich et al., 1996; Wang et al., 2009). To confirm results obtained by MS, RSP samples were analyzed by western blot using the gpE-specific antibody 4G2. Conditioned medium containing expressed RSP was centrifuged to pellet intact RSPs, separating RSP from soluble gpE and degraded particles. Western blot of resuspended pellets demonstrated gpE present at approximately 55 kD and 110 kD, indicating both monomeric and dimeric forms of gpE (Fig. 1D). The presence of dimeric gpE further supports the expression of gpE in particulate form, as gpE is present as a homodimer in mature virus and subviral particles (Kuhn et al., 2002). Additionally, the diffuse banding of gpE in western blot is consistent with the protein being glycosylated (Mondotte et al., 2007). Results obtained by TEM, MS and western blot were similar across RSPs of the four different strains with no significant differences. Overall, these results demonstrate RSPs are composed of the correct protein composition.

To determine RSP expression of the different strains, quantification of RSPs was performed by western blot using purified recombinant gpE as a standard. RSP expression levels for the different strains ranged from 0.6 to 6 μg of gpE monomer equivalence per mL of culture supernatant (Fig. 1E). While expression levels across multiple transfections varied little for individual RSP strains, expression between strains of RSP varied as much as 10-fold, suggesting the particular sequences may have a direct role in the expression and assembly efficiency of RSPs. Collectively, analyses by ultracentrifugation, TEM, MALDI-MS, and western blot all support that expressed RSPs are of the correct size and composition.

### 3.3. RSP binding to HS by ELISA

**Fig. 2:**
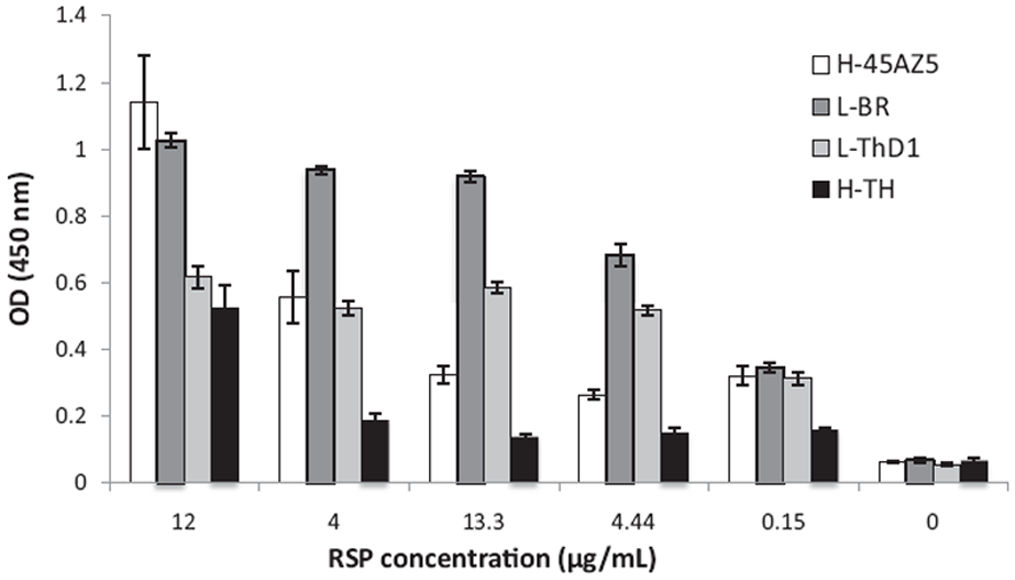
RSP interaction with heparin by ELISA Maxisorp plates coated with purified heparin–BSA were used to assess heparin binding of DV RSPs. Various concentrations of RSPs were used to show a dose response interaction. Error bars represent standard deviation.

Characterized RSPs of both clinical isolates and cell culture-adapted strains were then tested for their ability to recognize HS, first by a heparin ELISA method, which has been previously utilized for interrogating HS–virus interactions in a number of studies (Kato et al., 2010; Marks et al., 2001; Pattnaik et al., 2007; Watterson et al., 2012). Importantly, the assay incorporates heparin in an immobilized format, and thus heparin is presented in a similar manner as on the surface of cells. In a dose-dependent manner, RSPs were tested for their ability to bind heparin conjugated to BSA which was passively immobilized on microtiter plates. All RSPs showed significant binding above background at concentrations as low as 148 nM gpE equivalence in a dose-dependent manner (Fig. 2). The binding patterns varied among different virus strains, with L-ThD1 and L-BR strains (low-passage strains) reaching saturation at 1.3 μM gpE whereas the high-passage strains did not reach saturation at the highest concentration of 12 μM gpE. These results indicate that both clinical and highly passaged strains bind heparin, and surprisingly, highly passaged strains H-45AZ5 and H-TH showed lower binding efficiency than clinical strain RSPs.

### 3.4. Effect of soluble GAGs on RSPs binding to heparin on heparin-ELISA

While many studies characterizing DV–glycan interactions did not test both heparin and HS, some studies found that DV preferentially interacts with heparin with little or no binding to HS (Chen et al., 1997; Marks et al., 2001). Heparin is a highly sulfated form of HS and its expression is restricted to mast cells in connective tissues, whereas HS is ubiquitously found on most mammalian cell surfaces. We therefore sought to characterize the specificity of RSP binding to GAGs more broadly, including HS.

Heparin binding ELISA experiments were carried out with the modification that RSPs were first incubated with different GAGs in solution, allowing them to compete for RSP–heparin interaction (see Section 2). Heparin, HS, chondroitin sulfate A (CSA), and hyaluronic acid (HA) were included as soluble GAGs in competition studies. As anticipated, soluble heparin was able to effectively compete binding of all RSP strains to immobilized heparin, a finding that also supports the interaction being specific to heparin and not involving BSA (Table 1). Heparin was the most potent competitor followed by HS. Conversely, CSA and HA showed no competition despite using glycan concentrations up to 1 mg/mL. Additionally, H-45AZ5 and H-TH required lower concentrations of heparin and HS (~10-fold) than the low-passage strain RSPs to reach their half maximal inhibition concentration, indicating a potential greater affinity of L-BR and L-ThD1 to heparin than that of H-45AZ5 and H-TH strains. The results indicate that RSPs of both low- and high-passage strains bind specifically to heparin and HS, but not HA and CSA. Interestingly, these results also suggest that electrostatic charge from sulfate groups (absent in HA) and carbohydrate backbone (*i.e.*, heparin and HS have different disaccharide backbone than CS) may be important structural factors contributing to binding specificity, a finding consistent with other HS-binding viruses (Chen et al., 1997; Hilgard and Stockert, 2000; Kato et al., 2010; Marks et al., 2001; Watterson et al., 2012).

### 3.5. Effect of soluble heparin/HS on RSP-Vero cells binding

**Fig. 3:**
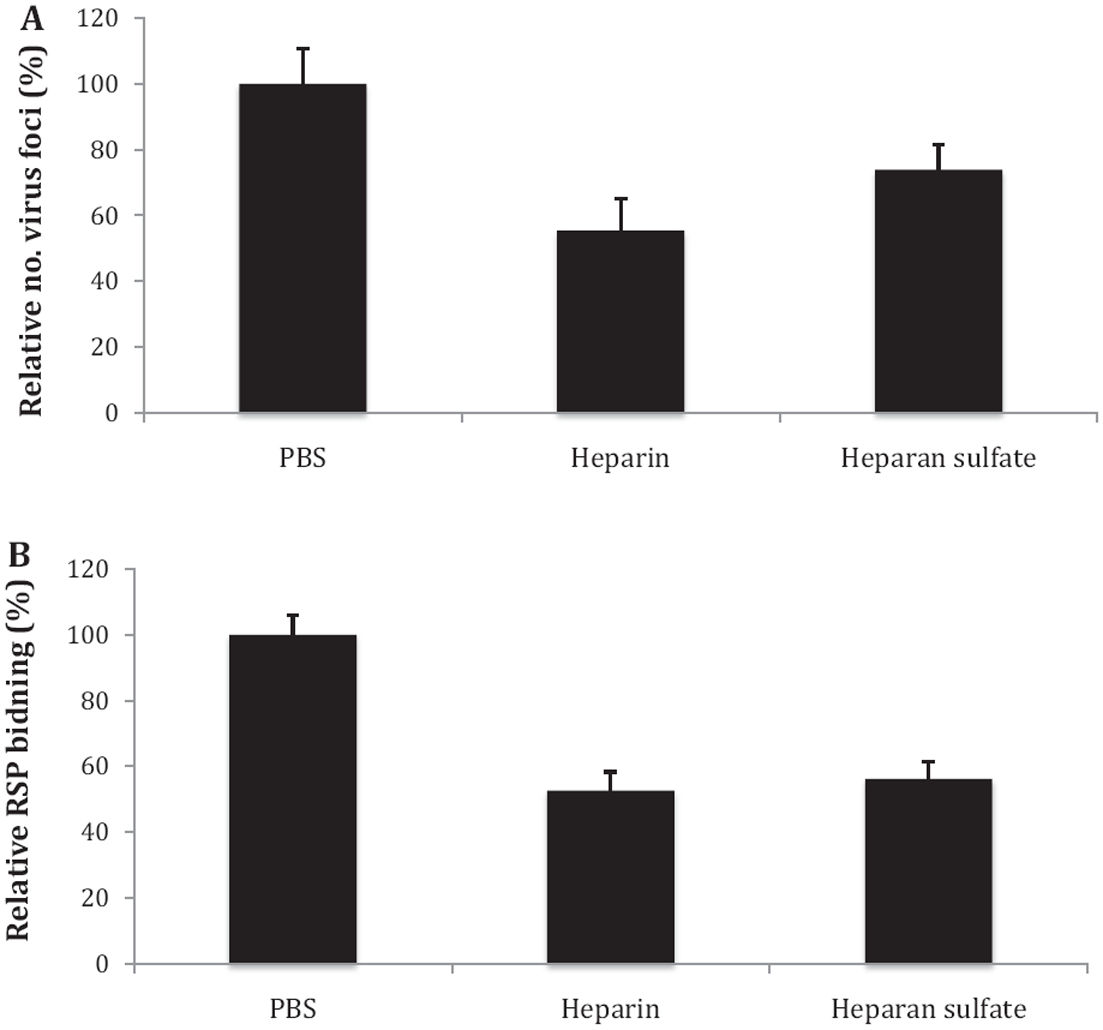
GAG inhibition of strain TH-Sman of infectious DV and RSP (A) Infectious DV TH-Sman (50 ffu/well) was incubated with heparin, HS or PBS for 2 h at 37 °C. The mixture was then applied to Vero cells in 24 well–well plates and incubated for 5 days. Foci were visualized after fixing by probing with anti-gpE antibody. Infected cells are reported as a percentage relative to PBS as a negative control. (B) Purified TH-Sman RSP was incubated with DMEM/F12 medium containing heparin, HS or PBS as a negative control. After 2 h incubation, samples were added to Vero cell monolayers seeded in 96-well plates and incubated for 4 h at 37 °C to allow RSP binding to cells. Cells were then fixed and bound RSP was detected by anti-gpE antibody. Error bars represent standard deviation.

To further investigate the involvement of HS as an attachment factor for DV, binding assays were performed with cells, which better capture the natural diversity and presentation of glycans that DV would encounter compared to ELISA-based methods. To determine whether RSP binding to cells would capture key elements of infectious DV binding to cells, initial cell-binding control experiments were performed with both RSP and infectious DV of the same strain, TH-Sman. Heparin and HS were able to similarly inhibit both RSP and DV binding to cells (Fig. 3). These results support the notion that RSPs have similar gpE presentation as infectious virus to interact with receptors on target cells, a finding consistent with previous studies (Corver et al., 2000; Schalich et al., 1996).

**Fig. 4:**
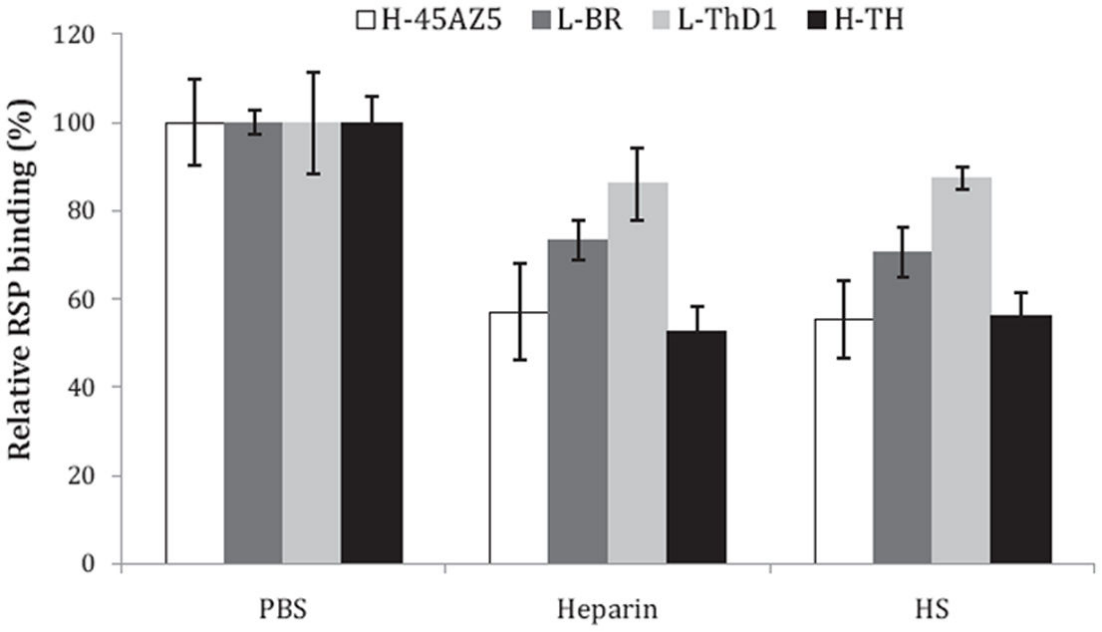
GAG inhibition of RSP binding to Vero cells Purified RSPs were incubated with DMEM/F12 medium containing heparin, heparan sulfate or PBS as a negative control. After 2 h incubation, samples were added to Vero cell monolayers seeded in 96-well plates and incubated for 4 h at 37 °C to allow RSP binding to cells. Cells were then fixed and bound RSPs were detected by anti-gpE antibody. Error bars represent standard deviation.

To test all strains, RSPs were incubated with medium containing heparin, HS, or no GAG addition (as control), after which the mixtures were applied to Vero cell monolayers. Both soluble heparin and HS at 0.1 mg/mL significantly inhibited RSPs-Vero cell binding for all four strains. For RSP of H-TH, for example, inhibition was as low as 47% for heparin and 44% for HS compared to no treatment control group (Fig. 4). The inhibitory potency between heparin and HS for each strain in this assay is not significantly different. These results indicate both clinical and high-passage strain RSPs bind HS on cells and are consistent with previous ELISA results demonstrating heparin and HS binding of all strains.

## 4. Discussion

This study was motivated by the question of whether HS is used as an attachment factor during infection of clinical strains of DV. Multiple previous studies have shown adaptation of DV by gaining of a positive charge substitution in gpE after continuously passaging in cell culture. As a result, these DV strains demonstrate an augmented HS-binding phenotype (Anez et al., 2009; Lee et al., 2006b; Prestwood et al., 2008). This study utilized RSP technology as a new approach to investigate the use of HS as a (co-)receptor for DV. RSPs have similar presentation of gpE to that of dengue virions, and we show that RSP interactions with Vero cells share similar key features of heparin and HS sensitivity as infectious DV (Fig. 3). Utilizing RSPs enables the direct study of HS-interaction by biochemical methods (*e.g.*, ELISA) as well as cell-binding assays without concerns of inadvertently introducing adaptive mutations, which can and has reported to occur with infectious DV propagation (Anez et al., 2009; Lee et al., 2006b; Prestwood et al., 2008). In this study, four strains of DV1 representing low- and high-passage histories were selected for production of RSPs with their native, codon-optimized prM–gpE sequences. Western blot, EM, MALDI-MS, and separation profiles from sucrose gradient ultracentrifugation were used to examine the identity and integrity of the RSPs. Taken together, the results demonstrated the RSPs to be intact with similar physicochemical features as shown in previous studies (Allison et al., 2003; Wang et al., 2009).

Interaction between HS and DV has been demonstrated in previous studies by multiple assays, including ELISA and chromatography with immobilized heparin (Chen et al., 1997; Kato et al., 2010; Lee et al., 2006b; Marks et al., 2001; Pattnaik et al., 2007; Watterson et al., 2012), as well as changes in infectivity after treatment of cells with heparinases (Germi et al., 2002; Hilgard and Stockert, 2000; Martinez-Barragan and del Angel, 2001; Okamoto et al., 2011; Prestwood et al., 2008; Thaisomboonsuk et al., 2005; Thepparit et al., 2004) or presence of competing soluble heparin/HS (Chen et al., 1997; Germi et al., 2002; Hilgard and Stockert, 2000; Kato et al., 2010; Lee et al., 2006b; Lin et al., 2002; Okamoto et al., 2011; Thaisomboonsuk et al., 2005). We first studied HS–RSP interaction by use of a heparin ELISA, the results of which showed binding of all four strains of DV1 RSPs to immobilized heparin. The specificity of binding was demonstrated by competition with soluble heparin and HS. To confirm and extend these findings, *in vitro* cell-based studies were carried out. Soluble heparin and HS were found to potently inhibit of all four strains of RSPs to Vero cells as well as that of infectious DV strain TH-Sman. Collectively, these results are broadly consistent with previous studies showing interaction between DV and HS, and they extend the characterization DV–HS interactions by demonstrating the use of HS by clinically relevant strains.

Although all RSPs bound to heparin in ELISA studies, the intensity of binding across RSP concentrations varied for each strain. RSPs of L-BR and L-ThD1, strains which have low-passage histories, showed significant binding to heparin and the overall binding intensity appeared higher than that of RSPs of high passage strains. Our ELISA binding results, which showed greater interaction between heparin and the two RSPs of low-passage strains, was further supported by GAG competition results (Table 1). Competition studies demonstrated that, compared to high-passage strain RSPs, low-passage strain RSPs had relatively higher IC_50_ values for heparin and HS competition, indicating that these RSPs bound tighter to immobilized heparin. The pattern of greater interaction between RSPs and Vero cell was similarly shown by binding inhibition with competing heparin and HS (Fig. 4). These results support the conclusion that both low-passage and high-passage strains of DV1 utilize HS as an attachment factor.

Glycan specificity was investigated by the use of different GAGs as competitors to RSP binding to adsorbed heparin. Our results (Table 1) indicate that both primary sequence of the glycans as well as the extent of sulfation affect binding. Heparin and HS, which have similar backbone structures but different sulfation levels, specifically competed RSP binding to immobilized heparin and also partially inhibited RSP binding to Vero cells (Figs. 3 and 4). While other studies have found HS as necessary for DV binding and infection (Germi et al., 2002; Hilgard and Stockert, 2000; Martinez-Barragan and del Angel, 2001; Okamoto et al., 2011; Prestwood et al., 2008; Thaisomboonsuk et al., 2005; Thepparit et al., 2004), a few studies have shown that heparin and highly sulfated HS, but not unmodified HS, are able to bind DV/gpE and/or inhibit infection (Chen et al., 1997; Marks et al., 2001; Okamoto et al., 2011). Different strains and even different serotypes as well as different assay formats were used than with our study, which may lead to different observations.

Previous studies have shown that when propagated under certain cell culture conditions, multiple flaviviruses including DV2 and DV4 can undergo adaptive changes leading to an augmented HS-binding phenotype (Anez et al., 2009; Lee et al., 2006b; Prestwood et al., 2008). While multiple previous investigations of DV1, the subject of this study, have provided supporting evidence that DV1 does utilize HS as an attachment factor (Hilgard and Stockert, 2000; Kato et al., 2010; Thepparit et al., 2004), three studies have indicated the contrary (Lin et al., 2002; Okamoto et al., 2011; Talarico et al., 2005). In all cases, the strains used had either extensive passage histories or such information was not provided, leaving it unclear whether clinical, low-passage DV1 strains utilize HS. Herein, we have shown RSPs of both low- and high-passage strains of serotype 1 recognize HS and heparin. Apparent discrepant results observed between different studies may be explained by different assays and target cells used as well as strain-specific differences. Indeed, subtle to substantial strain-specific differences in HS-binding activities within a serotype have been observed (Lin et al., 2002; Okamoto et al., 2011). Additional viral and biochemical studies are needed to elucidate the mechanisms of HS usage by different strains and serotypes of DV.

In summary, we provide multiple lines of evidence supporting an interaction between HS and clinically relevant DV1 strains. This study was conducted using RSPs as a tool to capture properties of gpE presentation as occurs on native dengue virions without passaging of virus, which could lead to introduction of adaptive mutations. A number of HS-mimicking molecules have been characterized with anti-DV activity, namely fucoidan, carageenan, dl-galactan, and α-d-glucans (Alen and Schols, 2012; Hidari et al., 2008; Pujol et al., 2002; Qiu et al., 2007; Talarico and Damonte, 2007). PI-88, in particular, has been shown to increase survival in a mouse model of DV infection (Lee et al., 2006a). Without strong evidence that clinically relevant DV strains, in addition to labadapted strains, recognize HS, it remains unknown whether HS analogs would be effective in clinical settings. Our study directly demonstrates HS binding by both clinical and highly passaged strains of DV, and as such, further substantiates efforts to develop HS-mimicking molecules as potential antiviral agents for clinical treatment of dengue. Our results also provide evidence that dengue RSPs are similar to infectious dengue virions in their ability to recognize HS, a finding which supports the potential development of RSPs, having similar antigenic properties as dengue virions, as a safe and effective vaccine strategy (Ohtaki et al., 2010; Zhang et al., 2011). Taken together, the improved understanding of the molecular events of the DV infection process, including recognition of receptors, should aid the development of more specific and effective agents for combating dengue.

## Supplementary Material

Supplementary Figure 1

Supplementary Figure 2

Supplementary Table 1

## Figures and Tables

**Table 1:** RSP binding specificity probed by heparin ELISA with various competing GAGs.

RSP strain	IC_50_(μg/mL)			
	Heparin	Heparan sulfate	Chondroitin sulfate A	Hyaluronic acid
H-45AZ5	0.01	0.39	>10,000	>10,000
L-ThD1-0102	0.66	66.00	>10,000	>10,000
L-BR-DF02	0.54	47.70	>10,000	>10,000
H TH-Sman	0.01	0.15	> 10,000	>10,000
