# Supplementary material for: Recognition of heparan sulfate by clinical strains of dengue virus serotype 1 using recombinant subviral particles
Source: Virus Res. Author manuscript; Available in PMC 2014 Sep 1. (PMC4145673; doi:10.1016/j.virusres.2013.04.017)
Supplement: Supplementary Figure 2 [file NIHMS585329-supplement-Supplementary_Figure_2.docx]

**Supplemental Figure 2.**
