# Supplementary material for: Recognition of heparan sulfate by clinical strains of dengue virus serotype 1 using recombinant subviral particles
Source: Virus Res. Author manuscript; Available in PMC 2014 Sep 1. (PMC4145673; doi:10.1016/j.virusres.2013.04.017)
Supplement: Supplementary Table 1 [file NIHMS585329-supplement-Supplementary_Table_1.docx]

**Supplemental Table 1**. List of strains used in the study and passage history.

| **Strain name** | **Accession number** | **Low/High passage** | **Passage history** |
| --- | --- | --- | --- |
| 45AZ5 PDK-27 | AAB70695 | High | Originates from the serial passaging of the mild West Pac 74 (U88535) isolate in diploid fetal rhesus lung cell line with plaque cloning and mutagenesis; PDK-27 indicates further attenuation in PDK cells of original PDK-0 strain |
| ThD1-0102/01 | AY732479 | Low | From human serum |
| BR-DF02 | AB519681 | Low | Three passages in C6/36 cells |
| TH-Sman | D10513 | High | From patient serum then passaged 16 times in suckling mice followed by one time in C6/36 cells |
